# Transcriptome Analysis of circRNA and mRNA in Theca Cells during Follicular Development in Chickens

**DOI:** 10.3390/genes11050489

**Published:** 2020-04-29

**Authors:** Manman Shen, Ping Wu, Tingting Li, Pengfei Wu, Fuxiang Chen, Lan Chen, Kaizhou Xie, Jinyu Wang, Genxi Zhang

**Affiliations:** 1 College of Animal Science and Technology, Yangzhou University, Yangzhou 225009, China; mms@just.edu.cn (M.S.); d150070@yzu.edu.cn (T.L.); Wu_P_Fei@163.com (P.W.); fxchen1993@163.com (F.C.); chenlan9326@163.com (L.C.); yzxkz168@163.com (K.X.); zgx1588@126.com (G.Z.); 2 College of Biotechnology, Jiangsu University of Science and Technology, Zhenjiang 212003, China; wp4114@126.com

**Keywords:** theca layer, circular RNA, prehierarchical follicle, chicken, RNA-seq, egg performance

## Abstract

Development of ovarian follicles requires interactions between granulosa cells, theca cells, and oocytes. Multiple transcription levels are involved but information about the role of noncoding RNAs, especially circular RNAs (circRNAs), is lacking. Here, we used RNA sequencing to profile circRNAs and mRNAs in theca cells from three types of follicle: small yellow follicles (SYF), the smallest hierarchical follicles (F6), and the largest hierarchical follicles (F1). Using bioinformatics analysis, we identified a total of 14,502 circRNAs in all theca cells, with 5622 widely distributed in all stages of development. Differential expression analysis suggested that some genes display differential isoforms during follicular development. Kyoto Encyclopedia of Genes and Genomes (KEGG) analysis revealed enrichment of both differentially expressed circRNAs and mRNAs in pathways associated with reproduction, including the TGF-β signaling pathway, oocyte meiosis, and vascular smooth muscle contraction. Our study provides the first visual information about circRNAs and mRNAs in theca cells during follicle development in chickens and adds to the growing body of knowledge about theca cells.

## 1. Introduction

In domestic hens, successive growth of follicles during the reproduction period is needed to maintain the yield of eggs. Only a few follicles will grow to ovulation, and 95% of all follicles cease growth and undergo atresia. Once a follicle reaches a certain size (usually 6–8 mm in diameter in chickens), a biological process known as follicle selection is activated and follicles continue to grow in a hierarchical fashion, leading to ovulation [1,2].

Follicular walls consist of a mixture of somatic cells, including theca and granulosa cells. The effects of theca cells, granulosa cells, and oocytes on the development, differentiation, and apoptosis of follicles are mediated by a number of factors, including TGF-β family members, cAMP, and the StAR signaling pathway [3,4]. Previous studies have focused largely on granulosa cells since these can be easily isolated and grown in vitro [1]. The recent successful establishment of in vitro culture systems for avian theca cells has, however, allowed much needed studies on theca cell function [4,5]. It has been shown that the androgens and estrogens involved in development and apoptosis during follicular development are mainly synthesized by theca cells [5,6,7]. Follicular atresia in cattle appears to be closely associated with reduced thecal vascularity [8], and VEGF produced from granulosa cells after follicle selection has been shown to enhance angiogenesis within the thecal layer, which promotes yolk deposition in chickens [9]. Theca cells are thus key determinants of follicle development and selection, and elucidation of the mechanisms involved in theca cell progression could provide valuable insights into the mechanisms underlying follicle development.

In the human genome, only around 2% of the DNA is for coding genes and the remaining 98% produces noncoding RNAs [10]. Since their discovery by Salzman in 2012, circular RNAs (circRNAs) have attracted much research interest [11]. The expression of circRNAs is often tissue- and stage-specific and is highly conserved across eukaryotes [12]. Cyclization of RNA to give circRNA produces a stable structure that is not easily degraded by RNase R [13,14]. The major biological function of circRNAs is to act as miRNA sponges [15]. Advances in deep sequencing and bioinformatics techniques have allowed the identification of a large number of circRNAs in embryonic muscle and chicken livers [16,17]. Some circRNAs, such as circHIPK3 [18] and circSVIL [19], have been shown to play important roles in the growth of myoblast cells.

Information about the role of circRNAs in chickens is, however, sparse, especially with respect to follicle development. To address this, we have now systematically investigated the expression of circRNAs in chicken follicular theca cells. We used a strategy of second-generation sequencing to identify circRNAs in theca cells during follicle development in chickens. The findings can contribute to a better understanding of a catalog of circRNAs and mRNAs in chicken follicular development.

## 2. Materials and Methods

### 2.1. Ethics Statement

All experiments were approved by the Animal Care Committee of Yangzhou University, China (permit number SYXK (Su) 2012-0029). All experimental procedures were carried out in compliance with the Experimental Animals Protocols established by the Ministry of Science and Technology, and all efforts were made to alleviate suffering.

### 2.2. Harvesting of Theca Cells and RNA Isolation 

Sixteen generations of Jinghai Yellow chickens were reared by the Jiangsu Jinghai Poultry Industry Group Co., Ltd. (Nantong City, Jiangsu Province, China). During the laying period, the chickens were housed in individual cages under a 16 h light: 8 h dark regime, with 10% restriction of food and free access to water. Based on pedigree records, three half-sib hens with average body weight at 27 weeks were humanely sacrificed using 60%–70% carbon dioxide. Only hens with a soft eggshell egg in the oviduct were used in the study. The ovaries were removed and rinsed with PBS. Using Eresheim’s classification, the small yellow follicle (SYF, about 4–8 mm in diameter), the smallest hierarchical follicle (F6, about 9–12 mm in diameter), and the largest hierarchical follicle (F1, about 40 mm in diameter) were detached from the ovaries [20]. Theca cells were collected as described by Gilbert et al. [21] and frozen in liquid nitrogen as quickly as possible for downstream analysis.

Total RNA of nine samples from three individuals extracted using TRIzol reagent (Invitrogen, Carlsbad, CA, USA), RNA NanoDrop system (Thermo Fisher Scientific, Waltham, MA, USA), and Agilent 2100 Bioanalyzer Expert Software™ was used to measure the concentration and quality of total RNA, respectively. All the RNA samples with 28S/18S > 1, OD 260/280 in the range 1.8–2.1, and (RNA integrity number) RIN score > 9.0 were retained to generate sequenced samples.

### 2.3. Preparation of RNA Sequencing Libraries 

#### 2.3.1. mRNA Library Preparation

DNA and ribosomal RNA were removed to generate the mRNA sequencing (mRNA-seq) libraries. Sequencing libraries were generated using an rRNA-depleted RNA by NEBNext^®^ Ultra™ Directional RNA Library Prep Kit for Illumina^®^ (NEB, Ipswich, MA, USA), following the manufacturer’s recommendations. Products with 200–500 bp were purified and quantified, and library quality was assessed using an Agilent Bioanalyzer 2100 system (Agilent Technologies, Carlsbad, CA, USA). Library preparation and Illumina sequencing were performed by Novogene Co., Ltd. (Tianjin, China). Quality control was carried out using bioinformatics methods. The sequence information was mapped to the chicken reference genome GRCg6a (galGal 6.0) using Bowtie2 v2.2.8 [22] and HISAT2 v2.0.4 [23] software, and the results are shown in Table S1.

#### 2.3.2. circRNA Library Preparation

circRNA libraries of theca cells were constructed using mRNA-seq methods, except that linear RNA digested by 3U of RNase R (Epicentre, Madison, WI, USA) was added before assessment. The accession number of the sequencing data in the NCBI SRA database is PRJNA511712.

### 2.4. Transcript Identification and Feature Analysis

For quality control, raw data were firstly prepared in fastq format to remove reads containing adapter contaminants and poly-N. Q20, Q30, and GC scores were then calculated using the clean data (Table S1). A reference genome was then built using Bowtie2 v2.2.8 and paired-end clean reads were aligned to the reference genome using Bowtie [22]. Two methods of find_circ [24] and CIRI2 [25], were used to identify circRNAs. Overlap of these two methods provided the final novel circNRAs. The distribution of circRNAs in chromosomes was then calculated, and we sought to characterize the features of the identified circRNAs based on physical position in the genomic sequence and source genes. 

### 2.5. Differential Expression Analysis and Bioinformatics Analysis 

Differential expression analysis of transcripts from theca cells from two types of follicle was performed using the DESeq2 R package [26]. Adjusted *p*-values (*q*-values) were calculated to take into account the false discovery rate. Raw counts of mRNAs and circRNAs were first normalized using fragments per kilobase of exon model per million reads mapped (FPKM) and Transcripts Per Million (TPM), respectively. Transcripts showing fold changes ≥ 2, with *q*-values ≤ 0.05, were classified as differentially expressed. The results of these analyses were displayed graphically using the R software packages “pheatmap” [27] and “ggplot2” [28]. Biological processing terms from gene ontology (GO_BP) and Kyoto Encyclopedia of Genes and Genomes (KEGG) in DAVID (http://david.ncifcrf.gov/) databases were used for the differentially expressed mRNAs and the host genes of differentially expressed circRNAs.

### 2.6. Validation of circRNAs and mRNA

Divergent primers (Table S2) were designed according to the previously described splice sites [29] to produce PCR products of the circRNAs. PCR products were detected by agarose gel electrophoresis, and Sanger sequencing was carried out at Sango Biotech Co., Ltd. (Shanghai, China). Real-time fluorescent quantitative PCR (RT-qPCR) was used to confirm the expression patterns of the circRNAs. The program was carried out using an ABI 7500 Real-Time PCR System (Life Technologies, NY, USA) with AceQ qPCR SYBR Green Master Mix (Vazyme Biotech Co., Ltd., Nanjing, China) in a final volume of 20 µL. Each assay was performed in triplicate using the following cycling conditions: 30 s at 95 °C, followed by 40 cycles of 5 s at 95 °C and 34 s at 60 °C. The 2^−△△Ct^ method was used to compare gene expression, with β-actin as the reference gene.

### 2.7. Polymorphism and Genotyping of circRNAs Sharing Exons of RalGPS2

It has recently been shown that predicted miRNA-binding sites in circRNAs have decreased SNP density compared with flanking sequences and random sites [30]. Two circRNA isoforms, produced from RalGPS2 and sharing exons 2, 8, and 9 which showed differential expression in theca cells (Table S3 marked with red font), were genotyped by polymerase chain reaction-single strand conformational polymorphism (PCR-SSCP) and genomic DNA (gDNA) sequencing using the primers listed in Table S2. gDNA from the wing veins of Jinghai Yellow chickens (*n* = 403) and Jinmao Hua chickens (*n* = 322) was extracted using the standard phenol-chloroform method [31]. Detailed information about Jinmao Hua chickens and Jinghai Yellow chickens were provided in previous reports [32,33]. PCR products of each individual chicken were genotyped using PCR-SSCP. PCR products with different bank types were sequenced using the Sanger method at Sango Biotech Co., Ltd. (Shanghai, China).

## 3. Results 

### 3.1. Characteristics of circRNAs

**Figure 1:**
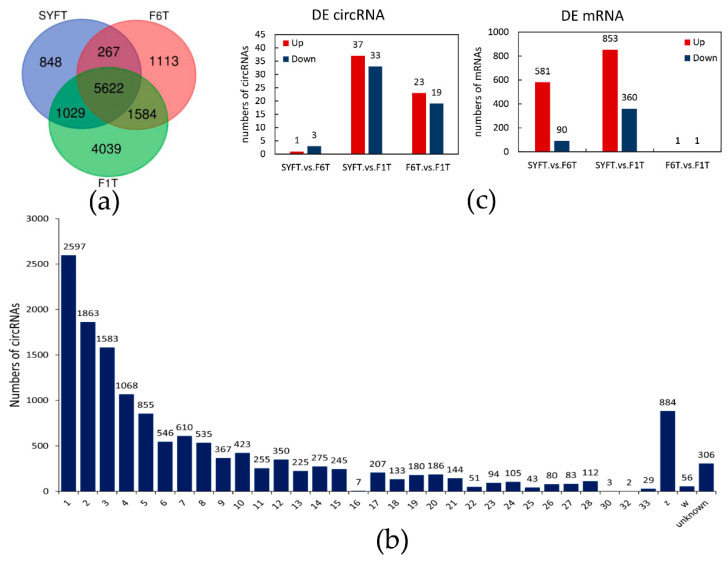
The novel detected circRNAs and differentially expressed analysis in theca cells: (**a**) Venn diagram of novel detected circRNAs in three types of theca cells; (**b**) distribution of circRNAs on the chromosome; and (**c**) the number of differentially expressed circRNAs and mRNAs. SYF: small yellow follicle, F6: smallest hierarchical follicle, F1: largest hierarchical follicle. T: theca cells.

Analysis of RNA, after ribosomal depletion and digestion of linear RNA, produced a total of 828,121,646 raw reads in nine circRNA libraries. After quality control, about 786,213,538 raw reads were produced, with a mean of 13.10 G clean bases. The mapping rate ranged from 92.3% to 97.3%. Among these mapped reads, the average GC content was 63.06% (Table S1). A total of 14,502 novel circRNAs were identified by two methods (Table S3). The average, minimum, maximum, and median sequence lengths were 274.5, 24, 1333, and 272 nt, respectively. Interestingly, as the thecal cells developed, more circRNAs were detected and 5622 circRNAs were found in theca cells from all three follicles (Figure 1a). A larger number was differentially expressed during follicular theca cells development. circRNAs were extensively distributed on chromosomes; by and large, the longer the chromosome the greater the number of splice-sites (Figure 1b). The features of the circRNAs are shown in Table S4. The majority of circRNAs had a length of about 100–500 nt, with most in the range 200–300 nt (Table S4a), and the genomic distance between splice-sites was typically about 1000–3000 bp (Table S4b). Intron and CDS regions produced almost the same number of circRNAs, and each accounted for approximately 40% of the total (Table S4c). Most circRNAs were produced from 1–4 exons, and about 40.4% of circRNAs was formed by two exons (Table S4d). Almost all of the host gene lengths were >8000 nt (Table S4e), and the majority of flank introns of circRNAs were >10^3^–10^5^ nt (Table S4f).

### 3.2. Differentially Expressed Transcripts and Functional Analysis 

**Figure 2:**
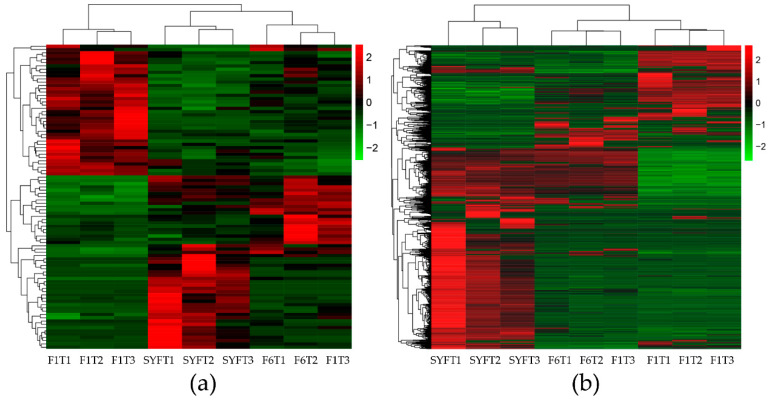
Heatmap of expression profiles for the differentially expressed circRNAs (**a**) and mRNAs (**b**). Each column displays one sample, and each row represents a circRNA or a mRNA. SYF: small yellow follicle, F6: smallest hierarchical follicle, F1: largest hierarchical follicle. T: theca cells.

Differentially expressed transcripts are shown in Figure 1c, and detailed information about these transcripts is provided in Tables S5 and S6. There was a total of 96 (10 duplicate circRNAs deleted) differentially expressed circRNAs in the three types of theca cells from the different follicles; 4, 70, and 42 in the theca cells of small yellow follicles (SYFT) vs. theca cells of smallest hierarchical follicles (F6T), SYFT vs. theca cells of largest hierarchical follicles (F1T), and F6T vs. F1T groups, respectively (Figure 1c and Table S5). The differentially expressed circRNAs were clustered according to their expression profiles (Figure 2). Samples at the same stages were clustered together for both circRNAs and mRNAs. circRNAs were first clustered in two groups, SYFTs and F6Ts, and then in F1Ts, while the mRNAs were first clustered in F6Ts and F1Ts and then in SYFTs. Notably, the expression patterns in F1Ts and SYFTs showed opposite trends.

Interestingly, the findings for differentially expressed mRNA were different. A total of 1886 mRNAs with 442 existing in both the SYFT vs. F6T and SYFT vs F1T groups and with 671 and 1213 in the SYFT vs. F6T and SYFT vs. F1T comparison groups, respectively, were found to be differentially expressed. Notably, only two mRNAs were differentially expressed in the F6T and F1T groups (Figure 1c and Table S6).

Analysis of the host genes of the differentially expressed circRNAs and mRNAs was carried out in the GO and KEGG databases using DAVID (Table 1 and Table 2). The biological process of GO (GO_BP) functions of the host genes of circRNAs included lipid localization, fatty acid transport, cell aging, and cell differentiation. The GO_BP functions of differentially expressed mRNAs included mainly cell cycle, cell growth, nuclear division, and reproductive processes. KEGG enrichment of differentially expressed circRNAs indicated involvement in pathways associated with reproduction. These included the VEGF, PPAR, TGF-β, and GnRH signaling pathways as well as oocyte meiosis. KEGG pathways of differentially expressed mRNAs were enriched in oocyte meiosis, ECM–receptor interaction, focal adhesion, and the cell cycle.

### 3.3. Relationship between circRNA and mRNA

**Figure 3:**
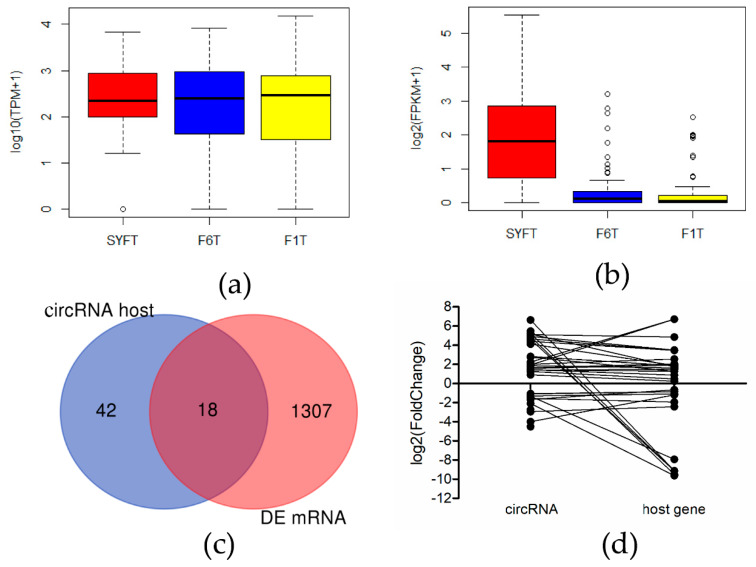
Relationship between circRNA and mRNA: (**a**) Expression level of differentially expressed mRNAs in three theca cells; (**b**) expression level of differentially expressed circRNAs in three theca cells; (**c**) Venn diagram of host genes of differentially expressed circRNAs and differentially expressed mRNAs; (**d**) the relationship of fold change between circRNAs and their host genes. SYF: small yellow follicle, F6: smallest hierarchical follicle, F1: largest hierarchical follicle. T: theca cells.

Expression levels of differentially expressed circRNAs, together with those of host genes of differentially expressed circRNAs and differentially expressed mRNAs were analyzed in the three types of theca cells (Figure 3). Interestingly, expression of mRNA in SYFT was higher than in F6T or F1T, whereas expression levels of circRNA were similar in all three types of cell. Eighteen genes (*THADA*, *SLCO5A1*, *MYH9*, *MAST2*, etc.) overlap between the host genes of differentially expressed circRNAs and differentially expressed mRNAs (Table S7). 

### 3.4. Validation of Experiments

**Figure 4:**
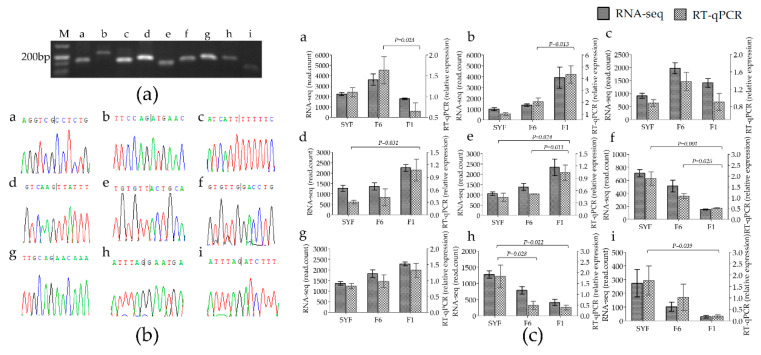
Validation of back-site and RNA-seq of circRNAs: (**a**) divergent primers amplify circRNAs in cDNA. (**b**) Sanger sequencing experimental validation the back-splicing site sequence of circRNA. (**c**) RT-qPCR validation of 9 differentially expressed circRNAs in three groups. SYF: small yellow follicle, F6: smallest hierarchical follicle, F1: largest hierarchical follicle. T: theca cell. Graph numbers of a, b, c, d, e, f, g, h, and i represent circRNAs chr2:75502|78826, chr23:5380525|5384728, chr4:49990652|50010410, chr3:31294378|31311114, chr2:47799748|47802307, chr3:40478181|40486533, chr3:76644181|76644976, chr8:6369673|6402248, and chr8:6369673|6422097, respectively.

A series of experiments, including PCR amplification, agarose gel electrophoresis, and Sanger sequencing, was performed to validate the back-splicing site of the nine circRNAs from all differentially expressed circRNAs (Figure 4). The specific divergent primers for circRNAs were all amplified PCR products (Figure 4a) and were validated by Sanger sequencing (Figure 4b). The RT-qPCR results also confirmed the tissue-specific expression of these circRNAs and the significant results were consistent with the RNA-seq data except chr4:49990652|50010410 and chr3:76644181|76644976 (Figure 4c), suggesting that our sequence data are reliable. Notably, two circRNAs, chr8:6369673|6402248 and chr8:6369673|6422097, were produced from the same three exons (exon 2, 8, and 9) of gene *RalGPS2,* with one or two different exons. 

### 3.5. SNP Mutation Detection

SNP polymorphisms in the target gene sequence, especially at miRNA-binding sites, could affect the regulation functionality. A single gene can produce multiple circRNAs [34], and we found that *RalGPS2* produced 14 circRNA isoforms, with two differentially expressed (*circRalGPS2_1* and *circRalGPS2_2* in Table S3 and Table S5 marked with red font) during theca cells development. The two isoforms share the same three exons, 2, 8, and 9. Approximately 78.6% (11/14) of the isoforms had exon2 (Table S3). By searching the reference genome, we found that three mutations may be distributed on the three exons of the two circRNAs isoforms generated from *RalGPS2* (*ENSGALG00000004273*). We performed PCR-SSCP for this region (Figure S1) but found none of the mutations in Jianghai Yellow chickens or Jinmao Hua chickens. 

## 4. Discussion

Folliculogenesis includes differentiation and proliferation of theca and granulosa cells and is controlled by numerous factors as well as transcriptional regulation. Progesterone is produced mainly by granulosa cells, whereas estrogen is produced by theca cells through the action of aromatase [35]. Some genes or proteins have been shown to be involved in important biological processes. For example, *CYP19A1* is more highly expressed in theca cells of pre-hierarchical follicles compared with theca cells of hierarchical follicles [36]. Clock genes, such as *BMAL1*, *PER2*, and *CLOCK*, are expressed in theca layers and are involved in steroidogenesis [37]. Studies investigating the function of circRNA in thecal cells are, however, lacking. To the best of our knowledge, this is the first report to profile circRNA expression in theca cells from chicken follicles. Because of our limited knowledge about their functions, circRNAs were once regarded as the “cross-talk” of the genome. With advances in sequencing technology and increased computational power, more and more circRNAs have been identified in a wide range of species. 

In the current study, we found both that circRNAs are generated specifically and not as cross-talk during transcription and that they show species diversity. For example, the GC content of circRNAs is 63.06%, which is higher than that of other noncoding RNAs, such as long noncoding RNA. This suggests specific production of circRNAs in thecal cells. The length of the majority of circRNAs is in the range 200–400 nt, the genomic region is <10 kb, and most circRNAs are generated from CDS and intron regions. The distribution feature is similar to that in the granulosa cells of follicles [38] and other chicken tissues [16,17]. 

Alternative splicing is a major contributory factor in the formation of circRNAs [39], and we found that 14 circRNA isoforms were generated from one gene, *RalGPS2*. miRNA-binding sites in circRNAs have been shown to have reduced SNP density [30], which is 10%–16% lower than for miRNA binding on mRNAs [40]. Our results are in agreement with the characteristics of miRNA target sites on mRNA, which suggests that hotspot exons may be more conserved to bind miRNAs. Unfortunately, binding sites between *circRalGPS2* and miR-200a-3p were not validated by a dual luciferase reporter gene assay in the present study. In our previous study, we showed that expression of *circRalGPS2* was highest in the ovarian stoma and lowest in the hierarchical follicle [38]. Further investigation into the function of *circRalGPS2* is needed.

Numbers and types of differentially expressed circRNAs and mRNAs were different, suggesting that they play different roles in transcriptional regulation and act through different mechanisms. mRNAs may be directly involved in life processes at the transcriptional level, whereas circRNAs can regulate mRNA expression at the posttranscriptional level [15]. circRNA can function in gene regulation by competing with linear splicing [41]. Yao et al. [42] found that *circZKSCAN1* and *ZKSCAN1* affect proliferation and invasion of hepatocellular carcinoma cells through different signaling pathways. In prostatic cancer, Chen et al. [43] found that some circRNAs produced from linear RNAs that are necessary for proliferation did not work by the same mechanism. These findings illustrate that the function of circRNAs is independent of host gene function. The number of upregulated differentially expressed mRNA molecules was higher in the SYFT vs. F6T group and expression of mRNAs was highest in SYFT. The SYF pool is heterogeneous [44] with >95% of SYFT undergoing atresia, which depends on various biological processes such as cellular differentiation, growth, apoptosis, and autophagy [2]. mRNAs are, therefore, most active in the SYFT stage. 

The heatmap results showed that the expression levels of circRNAs were first clustered in SYFTs and F6Ts, while mRNAs were first clustered in F6Ts and F1Ts. It has been proved that, the smaller the hierarchal follicular theca cells, the more estradiol they produce, while F1 follicle has lost the ability to convert progesterone to estradiol [45]. As the large yolky follicles mature, steroidogenesis shifts from the Δ5 to theΔ4 pathway [46]. The proliferation of theca cells (TCs) in F6 follicles is the beginning of a rapidly growing hierarchy [47] that is ready for yolk deposition via promoting angiogenesis [9]. Thus, the difference between the small follicular theca cells (SYFT and F6T) and the large follicular theca cells (F1T) is mainly the ability of estradiol produce. The difference between pre-hierarchal follicular theca cells (SYFT) and hierarchal follicular theca cells (F6T and F1T) is mainly the ability of yolk deposition. Based on the present study findings as well as the different follicular theca cells function, it can be concluded that the differentially expressed circRNA may be more closely related to maintaining estradiol concentration at the posttranscriptional level while the differentially expressed mRNAs may be correlated with yolk deposition at the transcriptional level.

Functional enrichment analysis showed that differentially expressed mRNA is mainly enriched in GO terms that include cell cycle, meiotic cell cycle, and nuclear division, suggesting that differentially expressed mRNAs are closely linked to the growth and differentiation of theca cells. KEGG analysis of differentially expressed mRNAs showed enrichment in reproduction pathways, including oocyte meiosis, ECM–receptor interaction, and focal adhesion. *IGF-I* and *BMPR2* were enriched in a wide range of pathways. *IGF-I*, which is present in both follicular granulosa and theca cells, affects steroid hormones, cellular proliferation, apoptosis, and follicle selection in either a paracrine or autocrine manner [48,49]. *BMPR2*, the gene for an important BMP family receptor, affects follicle growth via the Smad signaling pathway, which alters *BMPR1* phosphorylation induced by binding of BMP4, BMP7, and BMP15 to BMPR2 [50]. The expression of the two genes was, however, apparently higher in granulosa cells than in theca cells [50,51], illustrating that theca cells and granulosa cells are closely related. 

The KEGG pathways of differentially expressed circRNAs and mRNAs were the same and included the TGF-β signaling pathway, oocyte meiosis, and vascular smooth muscle contraction, which are all classical signaling pathways associated with follicle development. Previous studies showed that members of the TGF-β family, such as GDF9, BMPs, and AMH, play important roles in granulosa cell differentiation, proliferation, and steroidogenesis [52,53,54]. Oocyte-derived factor BMP15, which is confined to oocytes [50], has been shown to participate in oocyte meiosis in mice and sheep [55]. These findings, together with our own, highlight the complexity of biological processes, in which many pathways are interconnected and two or more types of transcripts orchestrate follicle development. Other KEGG pathways identified, including the VEGF and PPAR signaling pathways and metabolic pathways, were mainly associated with reproductive processes. VEGF enhances angiogenesis not only in the theca layer of primates [56] but also in that of birds [9]. During follicle selection, VEGF indirectly facilitates yolk deposition by promoting angiogenesis within the theca layer [9]. The numbers of blood vessels within the follicle theca layer before and after follicle selection were dramatically different [57], and an inadequate supply of blood vessels penetrating throughout the theca layer is associated with follicle atresia [8]. Capillaries extending from the follicle stroma surrounding the internal thecal layer provide a channel for nutrient transfer from the lumen to the perilumen gap [58]. Yolk precursors synthesized by the liver are transferred through theca cells, granulosa cells, and the zone radial band, with accumulation regulated by the lipoprotein receptor of the oocytes [59,60]. Optimal yolk deposition ensures that follicle development follows an orderly pattern [61], suggesting that theca cells are indispensable for yolk deposition. Immunocompetent cells, such as macrophages, dendritic cells, B cells, and T cells, are closely associated with theca cells, suggesting that theca cells play a role in immune function [62]. Activity of caspase-3 was higher in theca cells than in granulosa cells [63]. The transition from pre-hierarchical follicle to F1 follicle, which is a process of rapid follicle development, also requires a lot of energy [64]. Taking into account the biological function of theca cells and the KEGG enrichment of circRNAs, we conclude that circRNAs play important roles in metabolic and immune processes as well as in apoptosis, proliferation, and differentiation during the development of theca cells.

## Figures and Tables

**Table 1:** Gene ontology (GO) analysis for all differentially expressed transcripts in all groups.

^1^ GO _BP Analysis for Host Genes of ^2^DE circRNAs	*p*-Value	^1^ GO_BP Analysis of ^2^ DE mRNAs	*p*-Value
Negative regulation of cytokine-mediated signaling pathway	6.1 × 10^−2^	Nuclear division	1.6 × 10^−5^
Negative regulation of response to cytokine stimulus	6.3 × 10^−2^	Meiotic cell cycle	2.8 × 10^−5^
Lipid localization	7.1 × 10^−2^	Organelle fission	2.9 × 10^−5^
Anatomical structure homeostasis	7.4 × 10^−2^	Regulation of reproductive process	8.3 × 10^−5^
Activation of cysteine-type endopeptidase activity involved in apoptotic process	8.3 × 10^−2^	Meiotic cell cycle process	1.4 × 10^−4^
Fatty acid transport	1.0 × 10^−1^	Meiotic nuclear division	3.0 × 10^−4^
Cardiac muscle cell differentiation	1.1 × 10^−1^	Cell cycle process	3.5 × 10^−4^
Regulation of cytokine-mediated signaling pathway	1.1 × 10^−1^	Regulation of nuclear division	5.2 × 10^−4^
Regulation of response to cytokine stimulus	1.2 × 10^−1^	Mitotic nuclear division	5.9 × 10^−4^
Positive regulation of cysteine-type endopeptidase activity involved in apoptotic process	1.2 × 10^−1^	Regulation of cell cycle process	6.0 × 10^−4^
Regulation of cell proliferation	1.2 × 10^−1^	Cell cycle	1.1 × 10^−3^
Positive regulation of cysteine-type endopeptidase activity	1.3 × 10^−1^	Regulation of cell cycle	1.5 × 10^−3^
Positive regulation of hydrolase activity	1.3 × 10^−1^	Regulation of cell growth	1.7 × 10^−3^
Cell aging	1.3 × 10^−1^	Negative regulation of reproductive process	2.2 × 10–3
Intracellular signal transduction	1.4 × 10^−1^	Mitotic cell cycle process	2.2 × 10^−3^
Positive regulation of endopeptidase activity	1.4 × 10^−1^	Regulation of growth	2.5 × 10^−3^
Regulation of signal transduction	1.4 × 10^−1^	Endoderm formation	2.7 × 10^−3^
Positive regulation of peptidase activity	1.4 × 10^−1^	Positive regulation of cell cycle process	2.9 × 10^−3^
Cardiocyte differentiation	1.5 × 10^−1^	Positive regulation of cytokinesis	3.2 × 10^−3^
Organophosphate catabolic process	1.5 × 10^−1^	Endoderm development	3.4 × 10^−3^

^1^ GO_BP: biological processing terms from gene ontology; ^2^ DE: differentially expressed.

**Table 2:** Kyoto Encyclopedia of Genes and Genomes (KEGG) enrichment analysis for all differentially expressed transcripts in all groups.

DE ^1^ circRNA Host Genes KEGG ^2^ Analysis	*p*-Value	DE ^1^ mRNA KEGG ^2^ Analysis	*p*-Value
VEGF signaling pathway	1.1 × 10^−1^	Oocyte meiosis	2.3 × 10^−3^
PPAR signaling pathway	1.2 × 10^−1^	ECM–receptor interaction	7.8 × 10^−3^
TGF-beta signaling pathway	1.4 × 10^−1^	Focal adhesion	1.4 × 10^−2^
GnRH signaling pathway	1.5 × 10^−1^	Regulation of actin cytoskeleton	2.3 × 10^−2^
Oocyte meiosis	1.7 × 10^−1^	Hedgehog signaling pathway	4.8 × 10^−2^
Vascular smooth muscle contraction	1.9 × 10^−1^	Cell cycle	7.0 × 10^−2^
Ubiquitin-mediated proteolysis	2.3 × 10^−1^	Progesterone-mediated oocyte maturation	7.6 × 10^−2^
MAPK signaling pathway	3.6 × 10^−1^	Cardiac muscle contraction	8.8 × 10^−2^
Metabolic pathways	6.9 × 10^−1^	Neuroactive ligand–receptor interaction	9.6 × 10^−2^
		Basal transcription factors	1.8 × 10^−1^
		Glutathione metabolism	1.9 × 10^−1^
		FoxO signaling pathway	2.0 × 10^−1^
		Adrenergic signaling in cardiomyocytes	2.0 × 10^−1^
		Cytokine–cytokine receptor interaction	2.1 × 10^−1^
		p53 signaling pathway	2.2 × 10^−1^
		Phagosome	2.3 × 10^−1^
		Cell adhesion molecules (CAMs)	2.6 × 10^−1^
		Jak-STAT signaling pathway	3.1 × 10^−1^
		TGF-beta signaling pathway	3.4 × 10^−1^
		Vascular smooth muscle contraction	3.7 × 10^−1^

^1^ DE: differentially expressed; ^2^ KEGG: Kyoto Encyclopedia of Genes and Genomes.

## Supplementary Materials

The following are available online at https://www.mdpi.com/2073-4425/11/5/489/s1, Table S1: Quality control of RNA-seq data. Table S2: Primers used in current study. Table S3: Novel circRNA identification in theca cells. Table S4: Feature of sequencing date of circRNA. Table S5: Differentially expressed circRNAs in different groups. Table S6: Differentially expressed mRNAs in different groups. Table S7: The overlapping genes between host genes of differentially expressed circRNAs and differentially expressed mRNAs. Figure S1: Electrophoretic diagram of PCR-SSCP for three exons of RalGPS2.
